# A Micrometric Transformer: Compositional Nanoshell Transformation of Fe^3+^‐Trimesic‐Acid Complex with Concomitant Payload Release in Cell‐in‐Catalytic‐Shell Nanobiohybrids

**DOI:** 10.1002/advs.202306450

**Published:** 2023-10-31

**Authors:** Joohyouck Park, Nayoung Kim, Sang Yeong Han, Su Yeon Rhee, Duc Tai Nguyen, Hojae Lee, Insung S. Choi

**Affiliations:** ^1^ Center for Cell‐Encapsulation Research Department of Chemistry KAIST Daejeon 34141 Republic of Korea; ^2^ Department of Chemistry Hallym University Chuncheon 24252 Republic of Korea

**Keywords:** adaptation, metal‐organic complexes, nanoshells, payload release, single‐cell nanoencapsulation

## Abstract

Nanoencapsulation of living cells within artificial shells is a powerful approach for augmenting the inherent capacity of cells and enabling the acquisition of extrinsic functions. However, the current state of the field requires the development of nanoshells that can dynamically sense and adapt to environmental changes by undergoing transformations in form and composition. This paper reports the compositional transformation of an enzyme‐embedded nanoshell of Fe^3+^‐trimesic acid complex to an iron phosphate shell in phosphate‐containing media. The cytocompatible transformation allows the nanoshells to release functional molecules without loss of activities and biorecognition, while preserving the initial shell properties, such as cytoprotection. Demonstrations include the lysis and killing of *Escherichia coli* by lysozyme, and the secretion of interleukin‐2 by Jurkat T cells in response to paracrine stimulation by antibodies. This work on micrometric Transformers will benefit the creation of cell‐in‐shell nanobiohybrids that can interact with their surroundings in active and adaptive ways.

## Graphical abstract

Stimuli‐responsive, composition‐changeable nanoshells on individual living cells are demonstrated with shells of Fe^3+^‐trimesic acid complex in phosphate‐containing media. Concurrent payload release, along with the compositional transformation to Fe^3+^‐phosphate complex, enables active adaption to and manipulation of the surrounding environment, showcased by the lysis of *E. coli* and stimulation of Jurkat T cells.

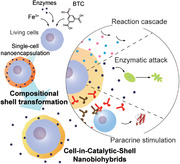

## 1 Introduction

Catalytic empowerment of living cells, in the form of cell‐in‐shell nanobiohybrids, provides an alternative to or more rapid and powerful tool for enhancement of cell's adaptive survival and functions than natural evolution or genetic engineering.^[^ ^1^, ^2^, ^3^, ^4^, ^5^ ^]^ In the field of single‐cell nanoencapsulation (SCNE) creating the cell‐in‐shell structures, cells have been armed with the ability for achieving exogenous catalytic reactions, not innate to the cells, by enzyme embedment in cytoprotective exoskeletal nanoshells and/or construction of inorganic nanozyme shells.^[^ ^6^, ^7^, ^8^ ^]^ Notable examples include the programmed functional switching of *Chlorella pyrenoidosa* from photosynthetic O_2_ production to photobiological H_2_ production, in which laccase on the polydopamine shell consumes O_2_ in the oxidation and second‐shell formation of tannic acid, generating an anaerobic microenvironment.^[^ ^9^ ^]^ In addition to the engineered whole‐cell biocatalysis and sensing,^[^ ^10^ ^]^ microbial and mammalian cells acquire self‐protective tools with the enzymes introduced into exoskeletal shells, exemplified by the enhanced survival of *Saccharomyces cerevisiae*, nanoencapsulated with β‐galactosidase‐MOF (MOF: metal‐organic framework) shells, in a nutrient‐deficient, lactose solution.^[^ ^11^ ^]^ As a related work, controlled degradation of protein‐embedded shells *on S. cerevisiae* has been coupled with drug‐delivery concept,^[^ ^12^ ^]^ and α_1_‐antitrypsin, released from MOF shells, protects the cells from hostile protease‐rich environments.^[^ ^13^ ^]^

**Figure 1:**
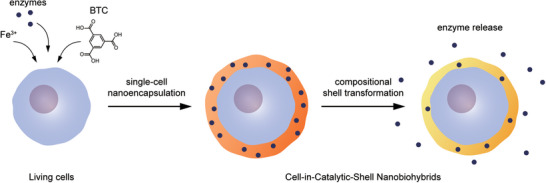
Conceptual scheme for micrometric Transformers in single‐cell nanoencapsulation, with enzyme‐embedded Fe^3+^‐BTC nanoshells as an illustrative example.

Advances in cell‐in‐shells have primarily been driven by the identification and development of shell materials and compositions. The utmost characteristic of the first‐generation shells, leading to realization of artificial spores,^[^ ^14^, ^15^ ^]^ was physicochemical durability, showcased by the shells of polydopamine,^[^ ^16^, ^17^, ^18^, ^19^ ^]^ silica,^[^ ^20^, ^21^, ^22^, ^23^, ^24^ ^]^ and titania,^[^ ^25^, ^26^ ^]^ enabling the effective cytoprotection against external aggressors.^[^ ^27^, ^28^ ^]^ Chemical control over shell degradation has made the second‐generation shells, generating micrometric Iron Men,^[^ ^29^ ^]^ whose shells are formed and broken apart in a controlled fashion. The eminent examples are the nanometric shells composed of MOFs^[^ ^30^, ^31^, ^32^, ^33^, ^34^, ^35^ ^]^ and Fe^3+^‐tannic acid complex.^[^ ^36^, ^37^, ^38^, ^39^, ^40^ ^]^ In particular, utilization of MOFs, or coordination complex in general, as shell materials has opened the door to the chemical empowerment of living cells, primarily because of enzyme stabilization in the structures.^[^ ^41^ ^]^ Collectively, the artificial shells that have so far been constructed can be regarded to be fixed in form and composition. In other words, shells are formed in a predesigned manner, and the static shells are degraded after fulfillment of their temporal roles, mostly cytoprotection, if needed. Next leap in the shell design lies in the fabrication of transformable or reconfigurable shells, which sense and respond and adapt to often abrupt environmental changes by transforming themselves in form and/or composition, not to mention resisting harmful conditions. This paper describes the compositional change of enzyme‐embedded shells, composed of supramolecular metal‐organic complex of Fe^3+^ and benzene‐1,3,5‐tricarboxylic acid (BTC, trimesic acid),^[^ ^42^, ^43^ ^]^ into shells of iron phosphate (Fe^3+^‐P), concomitant with enzyme release (**Figure** **1**). The transformed iron‐phosphate shells keep the ability for protecting the nanoencapsulated cells from various lethal stresses (e.g., heavy metals and lyticase), synergistically augmented by biochemical actions of the payloads released (e.g., enzymes) during their transformation.

We investigated phosphate anions as an inducer of compositional shell transformation in SCNE, leading to the creation of “micrometric Transformers”, with the consideration of their biological relevance and common presence in cell‐culture media. Phosphates are rich, after chloride and carbonate anions, in body fluids: for example, serum phosphate concentration is 0.83–1.63 mm in human adults.^[^ ^44^ ^]^ In addition, there has been much work on the MOF degradation (e.g., ZIF‐8 and MIL‐100(Fe)) in phosphate‐containing media.^[^ ^45^, ^46^ ^]^

## 2 Results and Discussion

**Figure 2:**
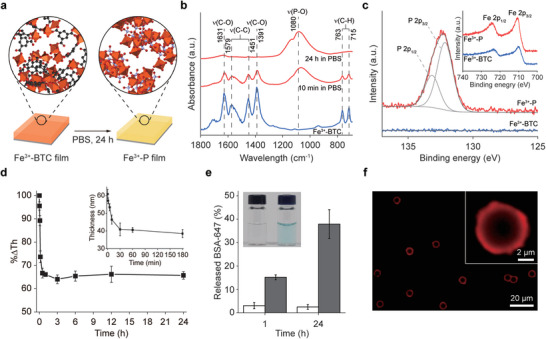
a–d) Compositional transformation of Fe^3+^‐BTC to Fe^3+^‐P films on Au: a) schematic for compositional transformation in PBS (pH 7.4), b) FT‐IR spectra of Fe^3+^‐BTC films (blue) before and (red) after incubation in PBS (for 10 min and 24 h), c) narrow‐scan P 2p XPS spectra of Fe^3+^‐BTC films (blue) before and (red) after incubation in PBS (for 24 h), inset: Fe 2p region, and d) graph of the remaining film‐thickness (%ΔTh) versus incubation time, inset: film thickness decrease during the first 3 h of incubation in PBS. e) Percentages of BSA‐647 released from PS[Fe^3+^‐BTC]_BSA‐647_, after incubation in (white) DI water and (gray) PBS (incubation time: 1 h and 24 h). Inset: optical photograph of the supernatants from PS[Fe^3+^‐BTC]_BSA‐647_, after incubation in (left) DI water and (right) PBS for 24 h. f) CLSM image of PS[Fe^3+^‐BTC]_BSA‐647_, after 24 h of incubation in PBS. Inset: a magnified image.

Prior to SCNE implementation, we investigated the compositional transformation of Fe^3+^‐BTC films in phosphate‐containing media, with non‐living objects as a model (**Figure** **2**a). We formed amorphous Fe^3+^‐BTC films on gold substrates, by following our reported protocol for material‐independent coating ([Fe^3+^] = 10 mm; [BTC] = 10 mm),^[^ ^42^ ^]^ and incubated them for 24 h in phosphate‐buffered saline (PBS, pH 7.4, [phosphate] = 4.02 mm). The film characterizations corroborated the transformation of Fe^3+^‐BTC to Fe^3+^‐P films. In the Fourier‐transform infrared (FT‐IR) spectra, the signature bands for BTC, such as carboxylic‐acid C‒O stretching (at 1631 and 1391 cm^−1^), aromatic‐ring C‒C stretching (at 1579 and 1451 cm^−1^) and aromatic‐ring C‒H out‐of‐plane bending (763 and 715 cm^−1^), disappeared after incubation in PBS, with appearance of a new band for phosphates at ≈1080 cm^−1^ (P‒O asymmetric stretching) (Figure 2b). The presence of phosphates in the transformed film was additionally supported by the X‐ray photoelectron spectroscopy (XPS) analysis that showed a P 2p peak at 132.3 eV, deconvoluted to peaks at 133.15 (P 2p_1/2_) and 132.15 eV (P 2p_3/2_), which was absent in the XPS spectrum of Fe^3+^‐BTC films (Figure 2c). The XPS peaks at 724.0 (Fe 2p_1/2_) and 710.8 eV (Fe 2p_3/2_) indicated that the Fe^3+^ ions remained in the films after incubation in PBS (Figure 2c, inset). Furthermore, the O 1s peak could be split to four peaks at 529.03 (Fe‒O‒Fe), 530.03 (Fe‒O‒P, P═O), 530.73 (P‒O‒H), and 531.73 eV (O‒C═O) (Figure S1, Supporting Information), confirming the transformation to Fe^3+^‐P. Meanwhile, the ellipsometric thickness measurement showed the concomitant thickness decrease with the compositional transformation: the film thickness decreased to 40.4 nm from 61.5 nm after 24 h of incubation in PBS (Figure 2d). The film thickness dropped sharply and plateaued afterward, implying that the transformation to Fe^3+^‐P was quite rapid. Correspondingly, the FT‐IR spectrum, taken after 3 h of incubation in PBS, showed the disappearance of the BTC peaks and appearance of the phosphate peak (Figure S2, Supporting Information).

Considering that the programmed release of payloads (e.g., enzymes) during the compositional film transformation would contribute to the cell bioaugmentation in a dynamic mode, we investigated protein release profiles from the shells formed on polystyrene (PS) particles (diameter: 5.07 µm) upon incubation in PBS, with Alexa Fluor 647‐conjugated bovine serum albumin (BSA‐647) as a probe protein. The core‐shell structures fabricated, denoted as PS[Fe^3+^‐BTC]_BSA‐647_, were incubated for 24 h in PBS. As a control, PS[Fe^3+^‐BTC]_BSA‐647_ was additionally incubated in deionized (DI) water. The BSA‐647 release during transformation was evident even to the naked eye (Figure 2e, inset), based on the color change of the supernatants to light blue, observed only for the sample in PBS. The UV–vis analysis showed 37.9% of BSA‐647 was released during 24 h of incubation in PBS, whereas the release was negligible in DI water (Figure 2e). The presence of BSA‐647 in the Fe^3+^‐P shell after transformation was supported by the confocal laser‐scanning microscopy (CLSM) images that clearly showed red rings around PS particles (Figure 2f).

**Figure 3:**
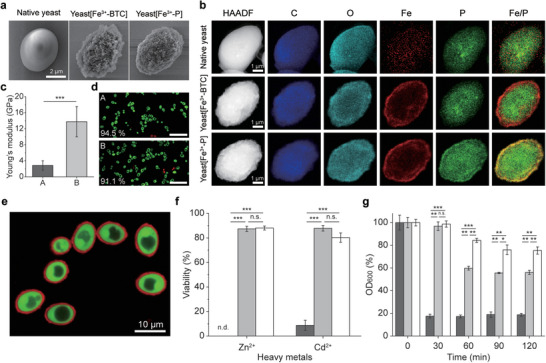
Construction and characterizations of yeast[Fe^3+^‐BTC] and yeast[Fe^3+^‐P]. a) FE‐SEM images of (left) native yeast, (middle) yeast[Fe^3+^‐BTC], and (right) yeast[Fe^3+^‐P]. b) HAADF‐STEM and corresponding EDS elemental mapping images of (top) native yeast, (middle) yeast[Fe^3+^‐BTC], and (bottom) yeast[Fe^3+^‐P]. c) Bar graph of Young's moduli of (A) yeast[Fe^3+^‐BTC] and (B) yeast[Fe^3+^‐P]. d) Relative viability of (A) yeast[Fe^3+^‐BTC] and (B) yeast[Fe^3+^‐P] with native yeast as a reference. Scale bar, 40 µm. e) CLSM images of yeast[Fe^3+^‐P]_BSA‐647_, after cell staining with FDA. f) Cytoprotection of (light gray) yeast[Fe^3+^‐BTC] and (white) yeast[Fe^3+^‐P] against Zn^2+^ and Cd^2+^. (gray) native yeast as control, n.d.: not detected. g) OD_600_ values of (gray) native yeast, (light gray) yeast[Fe^3+^‐BTC], and (white) yeast[Fe^3+^‐P], after incubation in a lyticase solution for the predetermined time. The statistical data are represented as mean ± SD (Student's *t*‐test; *n* = 20 for c; *n* = 4 for f; *n* = 3 for g; n.s.: not significant, *p < 0.05, **p < 0.01, ***p < 0.001).

After confirming the compositional transformation of Fe^3+^‐BTC to Fe^3+^‐P shells on PS particles, concomitant with payload release, we applied the reaction protocol to SCNE. *S. cerevisiae* (baker's yeast), as a representative single‐cell model, were encased within transformable, exoskeletal Fe^3+^‐BTC shells, creating Cell[Fe^3+^‐BTC] in general. We used yeast[Fe^3+^‐BTC] with five Fe^3+^‐BTC layers in this study, but no significant viability decrease was observed even after formation of seven Fe^3+^‐BTC layers (Figure S3a, Supporting Information). In addition, the viability of yeast[Fe^3+^‐BTC] was comparable to that of native yeast cells after 1 h of incubation at pH 2 (0.1 m HCl‐KCl buffer) and pH 10 (0.08 m glycine‐NaOH buffer) (Figure S3b, Supporting Information). After 24 h of incubation in PBS, yeast[Fe^3+^‐BTC] transformed into yeast[Fe^3+^‐P], indicated by noticeable changes in surface morphology: in the field‐emission scanning electron microscopy (FE‐SEM) images, the nanoparticulated, rough surfaces of yeast[Fe^3+^‐BTC] became smoothed after transformation (**Figure** **3**a). Further composition analysis was carried out by high‐angle annular dark‐field scanning transmission electron microscopy (HAADF‐STEM) imaging and corresponding energy‐dispersive X‐ray spectroscopy (EDS) elemental mapping (Figure 3b). The EDS images of yeast[Fe^3+^‐BTC] showed that the extrinsic Fe was accumulated on the outer surface of the cell, while biogenic P was distributed inside of the cell. The P intensity got weakened toward the edge, like the native, uncoated cell. In sharp contrast, after transformation to yeast[Fe^3+^‐P], the P intensity was uniformly distributed over the whole cell, and even a bright band was observed on the edge, where the extrinsic Fe co‐existed, indicative of the compositional shell transformation on *S. cerevisiae*. Changes in the zeta potential and the Young's modulus additionally supported the compositional transformation (Figure 3c; Figure S4, Supporting Information). Especially, the noticeable increase in the Young's modulus from 2.8 to 13.8 GPa after the compositional transformation indicated the hard characteristic of the Fe^3+^‐P shell (Figure 3c). The transformation protocol was applied to mammalian cells, such as HaCaT cells, as a proof‐of‐demonstration for expandability (Figure S5, Supporting Information). Although the viability of nanoencapsulated cells decreased from 84.7% to 50.8% after 24 h of incubation in PBS, the shell transformation was confirmed with EDS elemental mapping. It is notable that the viability of native HaCaT cells also decreased from 100% to 58.6% at a similar rate to the encapsulated cells, because PBS is a nutrient‐free medium and is not suitable for long‐term incubation of mammalian cells. We envision that exploration of optimized culture media would gently address the viability issue.

We observed no noticeable decrease in viability (3.4% decrease), after formation of the Fe^3+^‐P shell, based on the viability assay with fluorescein diacetate (FDA, green, for viable cells) and propidium iodide (PI, red, for dead cells), signifying the transformation conditions were cytocompatible (Figure 3d). In addition, longer incubation of 72 h in PBS did not cause significant viability loss (viability: 84.3%). The core‐shell structures of yeast[Fe^3+^‐P]_BSA‐647_ were clearly seen in the CLSM image, after staining viable cells with FDA (Figure 3e). The Fe^3+^‐P shell was also found to be degradable in response to external stimuli, such as ethylenediaminetetraacetic acid (EDTA) and ascorbic acid (Figure S6, Supporting Information). In addition, the cytoprotective capability of the shells were maintained against various harmful stressors. Yeast[Fe^3+^‐P] was incubated for 1 h in a 10 mm solution of Cd^2+^ or Zn^2+^, with yeast[Fe^3+^‐BTC] and native yeast as comparisons (Figure 3f). No viable cells were found for native yeast after incubation in the Zn^2+^ solution, whereas both yeast[Fe^3+^‐BTC] and yeast[Fe^3+^‐P] showed no noticeable decrease in viability. In the case of Cd^2+^, the viability decrease was 91.3% for native yeast, 12.1% for yeast[Fe^3+^‐BTC], and 19.8% for yeast[Fe^3+^‐P], respectively. The Fe^3+^‐P shell also rescued the cells from the lethal attack of lyticase, a cell‐wall‐degrading enzyme complex (Figure 3g). Under incubation with lyticase (0.2 mg mL^−1^) in the MES (2‐(*N*‐morpholino)ethanesulfonic acid) buffer (pH 7.4) for 2 h, native yeast showed a significant decrease in viability (viability: 18.8%). In contrast, the viability of yeast[Fe^3+^‐BTC] and yeast[Fe^3+^‐P] was found to be 56.1% and 75.4% respectively, indicating the enhanced protective capability of the Fe^3+^‐P shell. Taken all together, these results arguably verified that yeast[Fe^3+^‐BTC] transformed into yeast[Fe^3+^‐P] in a cytocompatible manner, while maintaining the crucial properties of artificial shells in SCNE, such as cytoprotection.

**Figure 4:**
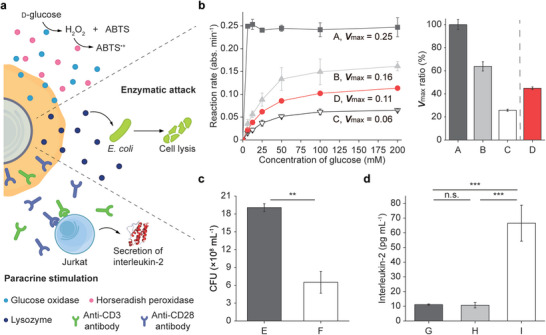
Dynamic cell bioaugmentation of micrometric Transformers. a) Schematic for dynamic cell bioaugmentation of micrometric Transformers: (top) GOx‐HRP cascade reaction of yeast[Fe^3+^‐BTC]_GOx_, (middle) lysis and killing of *E. coli* by lysozyme released during transformation of yeast[Fe^3+^‐BTC]_lysozyme_ to yeast[Fe^3+^‐P]_lysozyme_, and (bottom) IL‐2 secretion of Jurkat T cells upon paracrine stimulation by micrometric Transformers. b) (left) Michaelis‐Menten curves and *V*_max_ values for various GOx‐HRP reactions. A: free GOx; B: yeast[Fe^3+^‐BTC]_GOx_; C: yeast[Fe^3+^‐P]_GOx_; D: GOx released during transformation to yeast[Fe^3+^‐P]. (right) (red) Percentage of GOx released after 24 h of incubation in PBS. c) CFU values of *E. coli* after 24 h of co‐culture with (E) native yeast and (F) yeast[Fe^3+^‐BTC]_lysozyme_ in PBS. d) Amounts of IL‐2 secreted from (G) Jurkat T cells only, (H) co‐culture of Jurkat T cells with yeast[Fe^3+^‐BTC], and (I) co‐culture with yeast[Fe^3+^‐BTC]_anti‐CD3/anti‐CD28_ in RPMI 1640 medium for 24 h. The statistical data are represented as mean ± SD (Student's *t*‐test; *n* = 3 for c; *n* = 5 for d; n.s.: not significant, **p < 0.01, ***p < 0.001).

The active and programmed release of biologically functional molecules from artificial shells, in response to changes in the surrounding environment, would dynamically empower the cell‐in‐shell nanobiohybrids with the capability of adaptive survival and environment manipulation (**Figure** **4**a). We assessed the release of glucose oxidase (GOx) from the shells on *S. cerevisiae* by the ABTS (2,2′‐azino‐bis(3‐ethylbenzothiazoline‐6‐sulfonic acid) assay with horseradish peroxidase (HRP). The UV–vis analysis at 414 nm showed that 70.3% of GOx embedded in yeast[Fe^3+^‐BTC]_GOx_ was released in the aspect of enzyme activity after 24 h of incubation in PBS (Figure 4b). Furthermore, we could precisely control the release efficiency of the payload biomolecules by varying the shell thickness (Figure S7, Supporting Information). For example, the three‐layered shell could release its payload up to 90.3%. The model study with GOx indicated that a portion of enzymes could be released from micrometric Transformers, for acting designated roles in the media, while the transformed, cytoprotective Fe^3+^‐P shells remain armed with catalytic activities.

As a demonstration of environment manipulation by the released molecules, we constructed yeast[Fe^3+^‐BTC]_lysozyme_ and co‐cultured them with *Escherichia coli* for 24 h in PBS, in which lysozyme was released during compositional shell transformation. We first confirmed the killing effect of free lysozyme on *E. coli* under our experimental conditions (PBS, pH 7.4, for 24 h) (Figure S8, Supporting Information), because the optimal condition for lysis of *E. coli* by lysozyme was reported to be pH 8.6 and 30 mm of NaCl.^[^ ^47^ ^]^ The analysis of colony‐forming units (CFUs) showed that the CFU value was 19 × 10^8^ after co‐incubation of *E. coli* (initial OD_600_: 0.3) with native yeast. In stark contrast, co‐incubation with yeast[Fe^3+^‐BTC]_lysozyme_ noticeably decreased the CFU value to 6.5 × 10^8^ (Figure 4c), confirming the active role of the micrometric Transformer in environment manipulation. In addition, the Fe^3+^‐BTC‐based micrometric Transformer could serve as a controller of paracrine signaling by releasing signaling molecules. For example, Jurkat T cells secret interleukin‐2 (IL‐2) upon stimulation by anti‐CD3/anti‐CD28 monoclonal antibodies (mAbs),^[^ ^48^ ^]^ which could be controlled by payload release upon shell transformation. Accordingly, we constructed cell‐in‐shell structures that embedded the two mAbs, yeast[Fe^3+^‐BTC]_anti‐CD3/anti‐CD28_, and assessed their capability for activating Jurkat T cells in a paracrine fashion, by measuring the amount of IL‐2 released after 24 h of incubation in a Roswell Park Memorial Institute (RPMI) 1640 medium. The RPMI 1640 medium, containing 5.63 mm of phosphates, has commonly been used for culture of Jurkat T cells. As a control experiment, we investigated the effect of anti‐CD3/anti‐CD28 embedment on the shell properties, such as robustness, showing less retarded cell division upon the antibody embedment into the Fe^3+^‐BTC shell (Figure S9, Supporting Information). The enzyme‐linked immunosorbent assay (ELISA) showed that the baseline IL‐2 release, i.e., the amount of IL‐2 released by Jurkat T cells only, was ≈11 pg mL^−1^, which was not altered by the co‐incubation of Jurkat T cells with yeast[Fe^3+^‐BTC] that did not have any payloads in their shells. However, strikingly, paracrine recognition and signaling, made possible by yeast[Fe^3+^‐BTC]_anti‐CD3/anti‐CD28_, significantly increased the IL‐2 secretion by ≈600% to 67 pg mL^−1^ (Figure 4d). These examples confirmatively corroborated that Cell[Fe^3+^‐BTC], as micrometric Transformers, could act as an active and dynamic controller in various biochemical and biological setting in a multifarious manner.

## 3 Conclusion

The forefront field of cellular nanobiohybrids has striven to design and synthesize dynamic and adaptive cytohybrid structures that deviate from the conventional use of fixed, static nanoshells with predetermined shapes and configurations. These advanced structures are anticipated to effectively sense and respond to environmental changes, offering active and adaptive protection against harsh or lethal conditions while enabling versatile functionality, including environment manipulation. Our work represents the first demonstration of micrometric Transformers in this direction, in which Fe^3+^‐BTC nanoshells on cells transform compositionally to Fe^3+^‐P nanoshells with concomitant release of biologically active cargos. The catalytic activity of the enzymes liberated during the transformation process amplifies the ability of micrometric Transformers to safeguard the encapsulated cells from harmful stressors and manipulate the environment for their benefit. Embedding and releasing biomolecules under mild conditions also allows for the transportation of fragile biomolecules, such as antibodies, to target sites. In this context, our method would contribute to dynamic cell bioaugmentation that responds to and ameliorates the surrounding environment in a programmed manner, not to mention catalytic empowerment by coupling exogenous reactions with cellular metabolism. In addition, the use of phosphate anions as an inducer of compositional shell transformation enables micrometric Transformers to potentially serve as a cytotherapeutic agent for hyperphosphatemia by sequestering phosphates in the gastrointestinal tract and lowering systemic phosphate levels.^[^ ^49^ ^]^ We believe that our work could be beneficial in biotechnological and biomedical research, providing advanced tools for drug delivery systems and cell‐based therapies.^[^ ^50^, ^51^, ^52^, ^53^ ^]^

## 4 Experimental Section

### Single‐Cell Nanoencapsulation (SCNE)

A single colony of *S. cerevisiae* was picked from a yeast extract‐peptone‐dextrose (YPD) agar plate and cultured in a YPD broth with shaking at 30 °C for 30 h. To a pellet of *S. cerevisiae* were added sequentially 400 µL of the BTC stock solution and 400 µL of the Fe^3+^ stock solution. After gentle stirring for 1 min, the cells were washed with DI water. The process was repeated 5 times to produce yeast[Fe^3+^‐BTC]. Yeast[Fe^3+^‐BTC] were purified with centrifugation at 200 *g*, and suspended for 24 h in PBS for compositional transformation to Fe^3+^‐P. Cell viability was investigated with FDA and PI. The 5 µL of an FDA stock solution (10 mg mL^−1^ in acetone) and 2 µL of the PI stock solution (1 mg mL^−1^ in DI water) were mixed with 1 mL of a cell suspension for 20 min at 30 °C while shaking. The Fe^3+^‐BTC and Fe^3+^‐P shells were visualized after mixing 100 µL of an aqueous BSA‐647 solution (5 mg mL^−1^) with 900 µL of a cell suspension for 30 min at 30 °C while shaking. The cells were collected via centrifugation, washed with DI water, and analyzed by CLSM. For HAADF‐STEM imaging, cells were fixed with an aqueous solution of glutaraldehyde (2%) for 30 min and washed with DI water 3 times. After sequential dehydration with ethanol solutions (25%, 50%, 75%, 90%, 95%, 100%, 100%, and 100% (v/v) for 5 min each), a drop of diluted cell suspension was placed on a carbon‐supported copper grid (200 mesh) and dried overnight. For studies on the degradation of Fe^3+^‐P shells, a pellet of yeast[Fe^3+^‐P] was mixed with EDTA or ascorbic acid solution (100 mm) for 15 min, followed by washing with DI water 3 times. The resulting samples were subjected to the treatment with FDA and BSA‐647 and characterized by CLSM. For cytoprotection studies against heavy metals, cell pellets were incubated in an aqueous ZnCl_2_ or CdCl_2_ solution (10 mm) for 1 h and washed with DI water 3 times. The treated cells were subjected to the FDA/PI staining and analyzed by CLSM. To assess the capability of Fe^3+^‐BTC and Fe^3+^‐P shells to protect cells from lyticase‐mediated lethality, a lyticase stock solution was prepared by dissolving lyticase (2 mg) in a mixture of 500 µL of glycerol and 500 µL of an MES buffer (50 mm, pH 7.4). Native yeast, yeast[Fe^3+^‐BTC], or yeast[Fe^3+^‐P] were adjusted in cell density to an optical density of 0.5 at 600 nm (OD_600_) in an MES buffer that contained lyticase (0.2 mg mL^−1^). Cell viability was calculated based on OD_600_. The same SCNE protocol was applied to HaCaT cells for Fe^3+^‐BTC‐shell formation in an MES‐NaCl buffer (25 mm, 0.8% NaCl) (×3) and compositional transformation in PBS.

### Payload Release of Micrometric Transformers

a) *GOx‐HRP reactions*: To 400 µL of the BTC stock solution were added sequentially 5 µL of an aqueous GOx solution (1000 U mL^−1^) and 400 µL of the Fe^3+^ stock solution. The process was repeated 1, 3, or 5 times, leading to the formation of yeast[Fe^3+^‐BTC]_GOx_. The enzyme kinetics were analyzed at room temperature by the Michaelis‐Menten kinetics study. The assay solution was prepared by mixing 500 µL of a D‐glucose solution (400, 200, 100, 50, 25, or 12.5 mm), 10 µL of an aqueous HRP solution (25 U mL^−1^), and 100 µL of an ABTS solution (10 mm) in 190 µL of DI water (total volume: 800 µL). To the assay solution was added 200 µL of an aqueous GOx solution (1.25 U mL^−1^) or 200 µL of the supernatants combined from the shell‐forming processes. The maximum rate (*V*_max_) was estimated based on the UV–vis absorbance of ABTS^•+^ at 414 nm. The incorporation efficiency of GOx was calculated based on the *V*_max_ values for free GOx used for shell formation and yeast[Fe^3+^‐BTC]_GOx_. The shell transformation from Fe^3+^‐BTC to Fe^3+^‐P was carried out by incubating yeast[Fe^3+^‐BTC]_GOx_ for 24 h in PBS, and the supernatants were analyzed for enzyme kinetics. The percentage of released GOx during compositional transformation was calculated based on the *V*_max_ value for the supernatants. b) *Killing of E. coli by lysozyme*: To a pellet of *S. cerevisiae* were added sequentially 400 µL of the BTC stock solution, 20 µL of a lysozyme solution (10 mg mL^−1^), and 400 µL of the Fe^3+^ stock solution. After gentle stirring for 1 min, the cells were washed with DI water. The process was repeated 5 times, leading to the formation of yeast[Fe^3+^‐BTC]_lysozyme_. Native yeast, yeast[Fe^3+^‐BTC], or yeast[Fe^3+^‐BTC]_lysozyme_ were co‐cultured with *E. coli* for 24 h in PBS at 37 °C while shaking. The initial cell density of *E. coli* was set to an optical density of 0.3 at 600 nm (OD_600_) in PBS. After incubation, the harvested *E. coli* were plated onto Luria‐Bertani agar plates, and CFUs were assessed using a serial‐dilution method. (c) *Paracrine interactions and IL‐2 secretion of Jurkat T cells*: To a pellet of *S. cerevisiae* were added sequentially 100 µL of the BTC stock solution, 8 µL of anti‐CD3 mouse mAb (1 mg mL^−1^), 10 µL of anti‐CD28 mouse mAb (0.5 mg mL^−1^), and 100 µL of the Fe^3+^ stock solution. After gentle stirring for 1 min, the cells were washed with DI water. The process was repeated 5 times, leading to the formation of yeast[Fe^3+^‐BTC]_anti‐CD3/anti‐CD28_. The division characteristics of native yeast cells, yeast[Fe^3+^‐BTC], yeast[Fe^3+^‐P], yeast[Fe^3+^‐BTC]_anti‐CD3/anti‐CD28_, and yeast[Fe^3+^‐P]_anti‐CD3/anti‐CD28_ were investigated by measuring the OD_600_ values after incubation in a YPD broth for predetermined times. Cell density of native Jurkat T cells was set to 2.0 × 10^5^ cells mL^−1^ in the RPMI 1640 medium. Jurkat T cells were co‐incubated with yeast[Fe^3+^‐BTC]_anti‐CD3/anti‐CD28_ in 5 mL of the RPMI 1640 medium at 37 °C under 5% CO_2_. As a comparison, Jurkat T cells were co‐incubated with yeast[Fe^3+^‐BTC]. After 24 h of incubation, the cells were centrifuged, and the supernatants were collected and analyzed with the BD OptEIA – Human IL‐2 ELISA Kit II. The 50 µL of the ELISA Diluent and 100 µL of the supernatant were mixed in the microwells (6 wells per sample). After 2 h of incubation at room temperature, the wells were washed with the Washing Solution 5 times, and the Working Detector was added. After 1 h, the wells were washed with the Washing Solution 7 times, followed by addition of the TMB One‐Step Substrate Reagent (100 µL). After 30 min, 50 µL of the Stop Solution was added to each well, the absorbance of which was measured at 450 nm. Further experimental details are available on the experimental section of the Supporting Information.

### Statistical Analysis

The data were presented as mean values ± standard deviation. A comparison between two groups was analyzed using Student's *t*‐test. Statistical significance was assessed at a significance level (*α*) of 0.05 (*p < 0.05, **p < 0.01, ***p < 0.001, n.s.: not significant). The software programs of OriginPro 2019 and Microsoft Excel were utilized to perform the statistical analysis and create the graphs.

## Conflict of Interest

The authors declare no conflict of interest.

## Supporting information

Supporting Information

## Data Availability

The data that support the findings of this study are available from the corresponding author upon reasonable request.
