# Supplementary material for: A Micrometric Transformer: Compositional Nanoshell Transformation of Fe3+‐Trimesic‐Acid Complex with Concomitant Payload Release in Cell‐in‐Catalytic‐Shell Nanobiohybrids
Source: Adv Sci (Weinh). 2023 Oct 31;11(1):2306450. doi: 10.1002/advs.202306450 (PMC10767450; doi:10.1002/advs.202306450)
Supplement: Supplementary file 1 — Supporting Information [file ADVS-11-2306450-s001.pdf]

ADVANCED SCIENCE
Open Access

Supporting Information

for *Adv. Sci.*, DOI 10.1002/advs.202306450

A Micrometric Transformer: Compositional Nanoshell Transformation of Fe^{3+}-Trimesic-Acid Complex with Concomitant Payload Release in Cell-in-Catalytic-Shell Nanobiohybrids

Joohyouck Park, Nayoung Kim, Sang Yeong Han, Su Yeon Rhee, Duc Tai Nguyen, Hojae Lee and Insung S. Choi**

Supporting Information

A Micrometric Transformer: Compositional Nanoshell Transformation of Fe^{3+}-Trimesic-Acid Complex with Concomitant Payload Release in Cell-in-Catalytic-Shell Nanobiohybrids

Joohyouck Park, Nayoung Kim, Sang Yeong Han, Su Yeon Rhee, Duc Tai Nguyen, Hojae Lee, and Insung S. Choi**

Table of Contents

- Experimental Section.
- **Figure S1.** XPS spectra (O 1s) of Fe^{3+}-BTC and Fe^{3+}-P films on Au.
- **Figure S2.** FT-IR spectrum of Fe^{3+}-BTC films on Au, after 3h of incubation in PBS.
- **Figure S3.** (a) Viability of yeast[Fe^{3+}-BTC] with different numbers of Fe^{3+}-BTC layers. (b) Viability of native yeast cells and yeast[Fe^{3+}-BTC] after 1 h of incubation at pH 2 and 10.
- **Figure S4.** Zeta-potential changes of yeast after Fe^{3+}-BTC SCNE and compositional transformation to Fe^{3+}-P.
- **Figure S5.** SCNE of HaCaT cells.
- **Figure S6.** Shell degradation.
- **Figure S7.** Incorporation and release efficiencies of GOx with different numbers of Fe^{3+}-BTC layers.
- **Figure S8.** Lysis of *E. coli* by free lysozyme.
- **Figure S9.** Division profiles of native yeast cells, yeast[Fe^{3+}-BTC], (red) yeast[Fe^{3+}-P], yeast[Fe^{3+}-BTC]$_{anti\text{-}CD3/anti\text{-}CD28}$, and yeast[$Fe^{3+}$-P]$_{anti\text{-}CD3/anti\text{-}CD28}$.

Experimental Section

Materials. Benzene-1,3-5-tricarboxylic acid (trimesic acid, BTC, Sigma-Aldrich), iron(III) chloride hexahydrate ($FeCl_3 \cdot 6H_2O$, ≥98.0%, Sigma-Aldrich), phosphate-buffered saline (PBS, pH 7.4, Welgene), Alexa Fluor™ 647-conjugated bovine serum albumin (BSA-647, Thermo Fischer Scientific), ethylenediaminetetraacetic acid (EDTA, ≥98%, Sigma-Aldrich), L-ascorbic acid (≥99.0%, Sigma-Aldrich), D-(+)-glucose (≥99.5%, Sigma-Aldrich), glucose oxidase (GOx, from *Aspergillus niger*, Sigma-Aldrich), horseradish peroxidase (HRP, from *Amoracia rusticana*, Sigma-Aldrich), 2,2'-azino-bis(3-ethylbenzothiazoline-6-sulfonic acid) diammonium salt (ABTS, ≥98.0%, Sigma-Aldrich), glutaraldehyde solution (70% in H_2O, Sigma-Aldrich), ethanol (≥99.8%, Merck), acetone (≥99.8%, Merck), fluorescein diacetate (FDA, Sigma-Aldrich), propidium iodide (PI, Sigma-Aldrich), yeast-extract-peptone-dextrose broth (YPD broth, Duchefa Biochemistry), yeast-extract-peptone-dextrose agar (YPD agar, Duchefa Biochemistry), Luria-Bertani broth (LB broth, Duchefa Biochemistry), Luria-Bertani agar (LB agar, Duchefa Biochemistry), lysozyme (from chicken egg white, Sigma-Aldrich), lyticase (from *Arthrobacter luteus*, Sigma-Aldrich), zinc(II) chloride (99.999%, Sigma-Aldrich), cadmium(II) chloride (99.999%, Sigma-Aldrich), Jurkat T cells (Jurkat clone E6-1, No. 40152, Korean Cell Line Bank), Roswell Park Memorial Institute 1640 medium (RPMI 1640, with L-glutamine and 26 mM HEPES, Welgene), Dulbecco's modified Eagle's minimal essential medium (DMEM, with 4500 mg L^{-1} D-glucose, L-glutamine, 100 mg L^{-1} sodium pyruvate, and sodium bicarbonate, Welgene), monoclonal anti-CD3 antibody produced in mouse (UCHT1 to CD3e, Thermo Fisher Scientific), monoclonal anti-CD28 antibody produced in mouse (CD28.2, Thermo Fisher Scientific), trypan blue (0.4% solution, Thermo Fisher Scientific), fetal bovine serum (FBS, Welgene), penicillin-streptomycin (P/S, 5000 U mL^{-1} of penicillin and 5000 µg mL^{-1} of streptomycin, Welgene), BD OptEIA™ - Human IL-2 ELISA Kit II (BD Biosciences), polystyrene (PS) microparticles (diameter 5.07 µm, microparticles GmbH), and Si wafers (Sehyoung wafertech) were used as received. Gold (Au) substrates were prepared by thermal deposition of Ti (5 nm) and Au (100 nm) onto silicon wafers. Deionized (DI) water (18.3 MΩ·cm) from Milli-Q Direct 8 (Millipore) was used.

Characterizations. Polarized infrared external reflectance spectroscopy (PIERS) spectra were recorded with a nitrogen-purged Thermo Nicolet Nexus Fourier-transform infrared (FT-IR) spectrophotometer; IR spectra were equalized by adding approximately 2000 scans for background and each sample. X-ray photoelectron spectroscopy (XPS) spectra were recorded with a Sigma Probe (Thermo VG Scientific). Film thickness was measured with a spectroscopic ellipsometer Elli-SE (Ellipso Technology®). Field-emission scanning electron microscopy (FE-SEM) imaging was performed with an FEI Inspect F50 microscope (FEI) with an accelerating voltage of 10 kV, after sputter-coating with platinum. Enzyme kinetics, including release, were analyzed with a microplate reader (SpectraMax iD5, Molecular Devices). Confocal laser-scanning microscopy (CLSM) imaging was performed with an LSM 700 (Carl Zeiss). High-angle annular dark-field scanning transmission electron microscopy (HAADF-STEM) imaging and corresponding energy-dispersive X-ray spectroscopy (EDS) elemental mapping were performed with a Talos F200X (FEI) operated at 200 kV. Young's moduli were analyzed with a NanoWizard 4 XP Bioscience atomic force microscope (JPK).

Compositional Transformation of Fe^{3+}-BTC to Fe^{3+}-P. Stock solutions of BTC or Fe^{3+} were prepared by dissolving BTC or $FeCl_3$ in DI water to the final concentration of 10 mM. For formation of Fe^{3+}-BTC films on gold, gold substrates were immersed in 1 mL of the BTC stock solution, followed by addition of 1 mL of the Fe^{3+} stock solution. After 1 min of gentle stirring at 100 rpm, the gold substrates were washed with DI water. For formation of Fe^{3+}-

BTC nanoshells on PS particles, 300 μL of each stock solution were added to a pellet of PS particles made from 300 μL of a particle suspension in DI water (10% (w/v)). The process for film/shell formation was repeated 5 times. For compositional transformation to Fe^{3+}-P, the Fe^{3+}-BTC samples were incubated in PBS (pH 7.4, [phosphate] = 4.02 mM) for a predetermined time. For studies on payload release, to 300 μL of the BTC solution containing PS particles were added sequentially 6 μL of an aqueous BSA-647 solution (5 mg mL^{-1}) and 300 μL of the Fe^{3+} stock solution. The process was repeated 5 times, leading to the formation of PS[Fe^{3+}-BTC]$_{BSA-647}$. The amount of BSA-647 in the supernatants was assessed by measuring the UV-vis absorbance at 647 nm and used for the calculation of the amount of BSA-647 embedded in the Fe^{3+}-BTC shells of PS[Fe^{3+}-BTC]$_{BSA-647}$. After 24 h of incubation in PBS, the amount of BSA-647 released from PS[Fe^{3+}-BTC]$_{BSA-647}$ was calculated based on the UV-vis absorbance of the supernatants.

Single-Cell Nanoencapsulation (SCNE). A single colony of *S. cerevisiae* was picked from a yeast extract-peptone-dextrose (YPD) agar plate and cultured in a YPD broth with shaking at 30 °C for 30 h. To a pellet of *S. cerevisiae* were added sequentially 400 μL of the BTC stock solution and 400 μL of the Fe^{3+} stock solution. After gentle stirring for 1 min, the cells were washed with DI water. The process was repeated 5 times to produce yeast[Fe^{3+}-BTC]. Yeast[Fe^{3+}-BTC] were purified with centrifugation at 200 *g*, and suspended for 24 h in PBS for compositional transformation to Fe^{3+}-P. Cell viability was investigated with FDA and PI. The 5 μL of an FDA stock solution (10 mg mL^{-1} in acetone) and 2 μL of the PI stock solution (1 mg mL^{-1} in DI water) were mixed with 1 mL of a cell suspension for 20 min at 30 °C while shaking. The Fe^{3+}-BTC and Fe^{3+}-P shells were visualized after mixing 100 μL of an aqueous BSA-647 solution (5 mg mL^{-1}) with 900 μL of a cell suspension for 30 min at 30 °C while shaking. The cells were collected via centrifugation, washed with DI water, and analyzed by CLSM. For HAADF-STEM imaging, cells were fixed with an aqueous solution of glutaraldehyde (2%) for 30 min and washed with DI water 3 times. After sequential dehydration with ethanol solutions (25%, 50%, 75%, 90%, 95%, 100%, 100%, and 100% (v/v) for 5 min each), a drop of diluted cell suspension was placed on a carbon-supported copper grid (200 mesh) and dried overnight. For studies on the degradation of Fe^{3+}-P shells, a pellet of yeast[Fe^{3+}-P] was mixed with EDTA or ascorbic acid solution (100 mM) for 15 min, followed by washing with DI water 3 times. The resulting samples were subjected to the treatment with FDA and BSA-647 and characterized by CLSM. For cytoprotection studies against heavy metals, cell pellets were incubated in an aqueous $ZnCl_2$ or $CdCl_2$ solution (10 mM) for 1 h and washed with DI water 3 times. The treated cells were subjected to the FDA/PI staining and analyzed by CLSM. To assess the capability of Fe^{3+}-BTC and Fe^{3+}-P shells to protect cells from lyticase-mediated lethality, a lyticase stock solution was prepared by dissolving lyticase (2 mg) in a mixture of 500 μL of glycerol and 500 μL of an MES buffer (50 mM, pH 7.4). Native yeast, yeast[Fe^{3+}-BTC], or yeast[Fe^{3+}-P] were adjusted in cell density to an optical density of 0.5 at 600 nm (OD_{600}) in an MES buffer that contained lyticase (0.2 mg mL^{-1}). Cell viability was calculated based on OD_{600}. The same SCNE protocol was applied to HaCaT cells for Fe^{3+}-BTC-shell formation in an MES-NaCl buffer (25 mM, 0.8% NaCl) (×3) and compositional transformation in PBS.

Payload Release of Micrometric Transformers. *(a) GOx-HRP reactions:* To 400 μL of the BTC stock solution were added sequentially 5 μL of an aqueous GOx solution (1000 U mL^{-1}) and 400 μL of the Fe^{3+} stock solution. The process was repeated 1, 3, or 5 times, leading to the formation of yeast[Fe^{3+}-BTC]$_{GOx}$. The enzyme kinetics were analyzed at room temperature by the Michaelis-Menten kinetics study. The assay solution was prepared by mixing 500 μL of a D-glucose solution (400, 200, 100, 50, 25, or 12.5 mM), 10 μL of an aqueous HRP solution (25 U mL^{-1}), and 100 μL of an ABTS solution (10 mM) in 190 μL of

DI water (total volume: 800 μL). To the assay solution was added 200 μL of an aqueous GOx solution (1.25 U mL^{-1}) or 200 μL of the supernatants combined from the shell-forming processes. The maximum rate (V_{max}) was estimated based on the UV-vis absorbance of $ABTS^{\bullet +}$ at 414 nm. The incorporation efficiency of GOx was calculated based on the V_{max} values for free GOx used for shell formation and yeast[Fe^{3+}-BTC]$_{GOx}$. The shell transformation from Fe^{3+}-BTC to Fe^{3+}-P was carried out by incubating yeast[Fe^{3+}-BTC]$_{GOx}$ for 24 h in PBS, and the supernatants were analyzed for enzyme kinetics. The percentage of released GOx during compositional transformation was calculated based on the V_{max} value for the supernatants. *(b) Killing of E. coli by lysozyme:* To a pellet of *S. cerevisiae* were added sequentially 400 μL of the BTC stock solution, 20 μL of a lysozyme solution (10 mg mL^{-1}), and 400 μL of the Fe^{3+} stock solution. After gentle stirring for 1 min, the cells were washed with DI water. The process was repeated 5 times, leading to the formation of yeast[Fe^{3+}-BTC]$_{lysozyme}$. Native yeast, yeast[Fe^{3+}-BTC], or yeast[Fe^{3+}-BTC]$_{lysozyme}$ were co-cultured with *E. coli* for 24 h in PBS at 37 °C while shaking. The initial cell density of *E. coli* was set to an optical density of 0.3 at 600 nm (OD_{600}) in PBS. After incubation, the harvested *E. coli* were plated onto Luria-Bertani agar plates, and CFUs were assessed using a serial-dilution method. *(c) Paracrine interactions and IL-2 secretion of Jurkat T cells*: To a pellet of *S. cerevisiae* were added sequentially 100 μL of the BTC stock solution, 8 μL of anti-CD3 mouse mAb (1 mg mL^{-1}), 10 μL of anti-CD28 mouse mAb (0.5 mg mL^{-1}), and 100 μL of the Fe^{3+} stock solution. After gentle stirring for 1 min, the cells were washed with DI water. The process was repeated 5 times, leading to the formation of yeast[Fe^{3+}-BTC]$_{anti\text{-}CD3/anti\text{-}CD28}$. The division characteristics of native yeast cells, yeast[Fe^{3+}-BTC], yeast[Fe^{3+}-P], yeast[Fe^{3+}-BTC]$_{anti\text{-}CD3/anti\text{-}CD28}$, and yeast[$Fe^{3+}$-P]$_{anti\text{-}CD3/anti\text{-}CD28}$ were investigated by measuring the OD_{600} values after incubation in a YPD broth for predetermined times. Cell density of native Jurkat T cells was set to 2.0×10^5 cells mL^{-1} in the RPMI 1640 medium. Jurkat T cells were co-incubated with yeast[Fe^{3+}-BTC]$_{anti\text{-}CD3/anti\text{-}CD28}$ in 5 mL of the RPMI 1640 medium at 37 °C under 5% CO_2. As a comparison, Jurkat T cells were co-incubated with yeast[Fe^{3+}-BTC]. After 24 h of incubation, the cells were centrifuged, and the supernatants were collected and analyzed with the BD OptEIA™ - Human IL-2 ELISA Kit II. The 50 µL of the ELISA Diluent and 100 µL of the supernatant were mixed in the microwells (6 wells per sample). After 2 h of incubation at room temperature, the wells were washed with the Washing Solution 5 times, and the Working Detector was added. After 1 h, the wells were washed with the Washing Solution 7 times, followed by addition of the TMB One-Step Substrate Reagent (100 µL). After 30 min, 50 µL of the Stop Solution was added to each well, the absorbance of which was measured at 450 nm.

Statistical Analysis. The data are presented as mean values ± standard deviation. A comparison between two groups was analyzed using Student's *t*-test. Statistical significance was assessed at a significance level (α) of 0.05 (*$p < 0.05$, **$p < 0.01$, ***$p < 0.001$, n.s.: not significant). The software programs of OriginPro 2019 and Microsoft Excel were utilized to perform the statistical analysis and create the graphs.

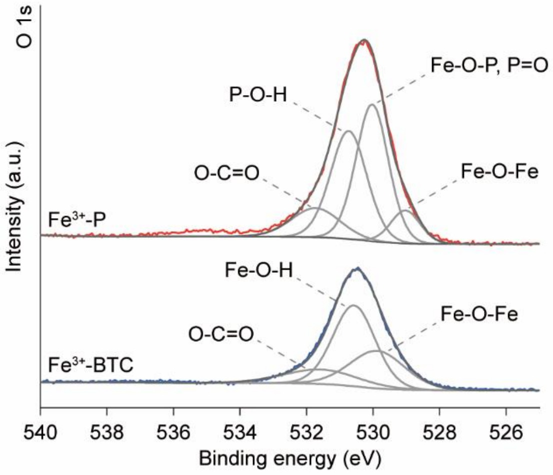

Figure S1. XPS spectra (O 1s) of Fe^{3+}-BTC and Fe^{3+}-P films on Au.

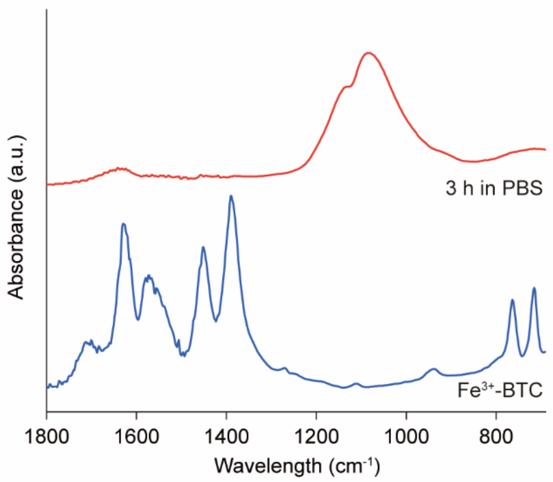

Figure S2. FT-IR spectrum of Fe^{3+}-BTC films on Au, after 3h of incubation in PBS.

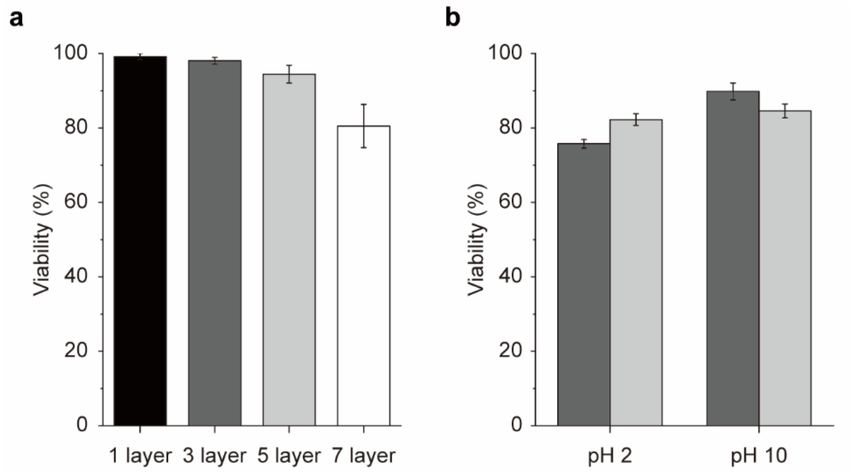

Figure S3. (a) Viability after formation of Fe^{3+}-BTC shells with different numbers of layers. (b) Viability of native yeast cells and yeast[Fe^{3+}-BTC] after 1 h of incubation at pH 2 and 10.

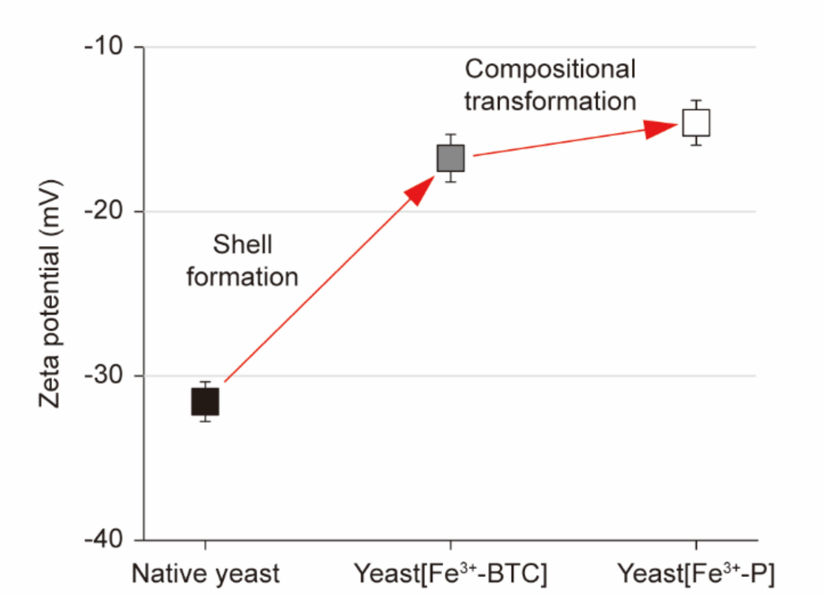

Figure S4. Zeta-potential changes of yeast after Fe^{3+}-BTC SCNE and compositional transformation to Fe^{3+}-P.

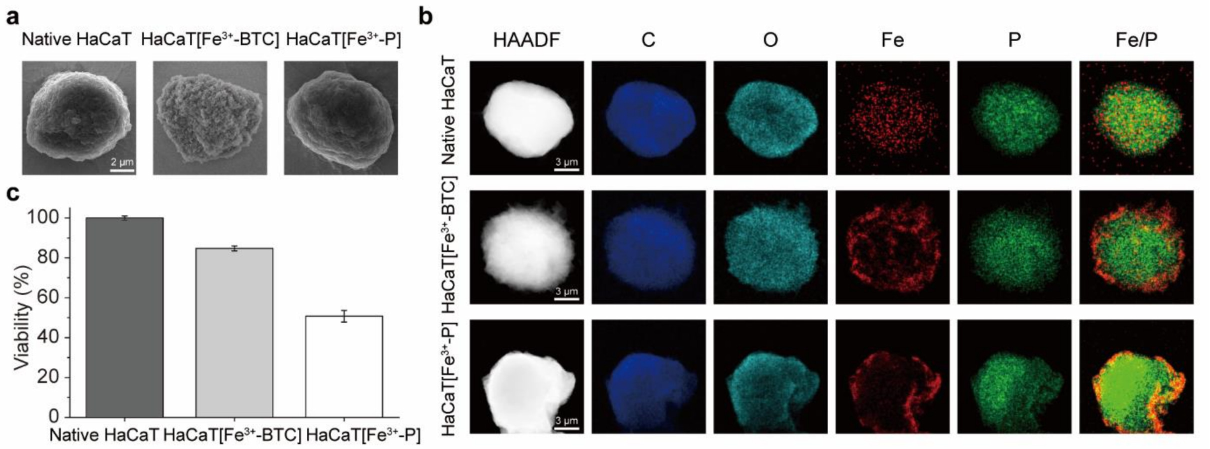

Figure S5. Construction and characterizations of HaCaT[Fe^{3+}-BTC] and HaCaT[Fe^{3+}-P]. a) FE-SEM images of (left) native HaCaT, (middle) HaCaT[Fe^{3+}-BTC], and (right) HaCaT[Fe^{3+}-P]. b) HAADF-STEM and corresponding EDS elemental mapping images of (top) native HaCaT, (middle) HaCaT[Fe^{3+}-BTC], and (bottom) HaCaT[Fe^{3+}-P]. c) Relative viability of HaCaT[Fe^{3+}-BTC] and HaCaT[Fe^{3+}-P] with native HaCaT as a reference. Relative viability of native HaCaT cells and HaCaT[Fe^{3+}-BTC] after 24 h of incubation in PBS: 58.6% and 50.8%.

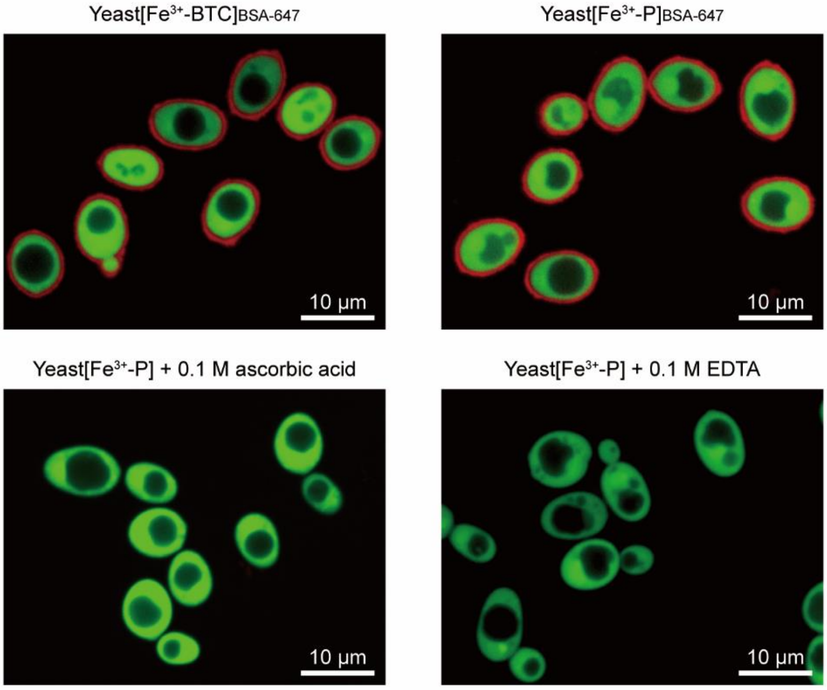

Figure S6. Shell degradation. CLSM images of yeast[Fe^{3+}-BTC], yeast[Fe^{3+}-P], 0.1 M ascorbic-acid-treated yeast[Fe^{3+}-P], and 0.1 M EDTA-treated yeast[Fe^{3+}-P]. Green: cells stained with FDA, red: shells labeled with Alexa Fluor™ 647-conjugated BSA.

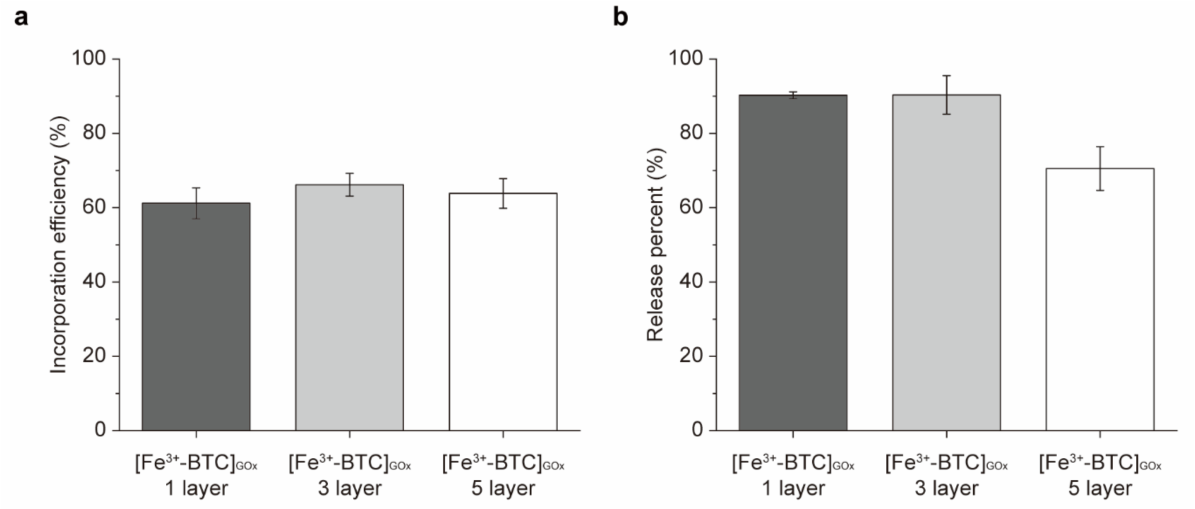

Figure S7. (a) Incorporation efficiency and (b) release percent of GOx with different numbers of Fe^{3+}-BTC layers on yeast cells (number of layers: 1, 3, and 5).

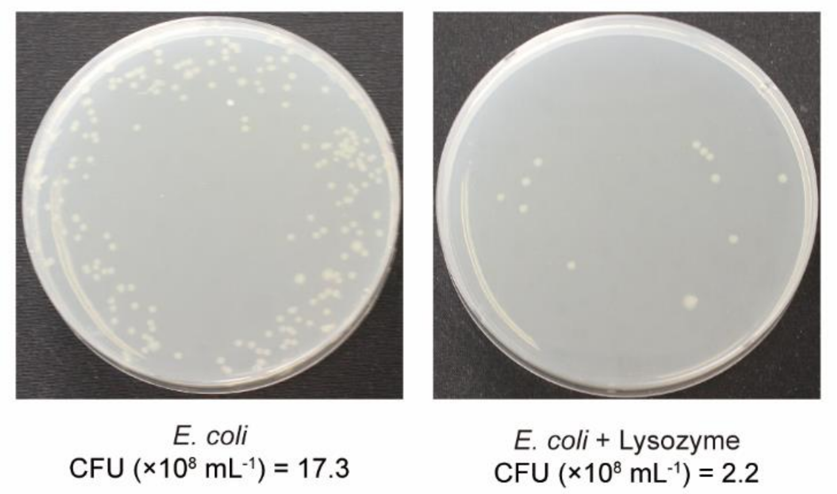

Figure S8. Lysis of *E. coli* by free lysozyme. Optical images and CFU values of *E. coli* and *E. coli* with lysozyme (1.0 mg mL^{-1}). OD_{600} of *E. coli*: 0.3. PS particles were added to the cultures.

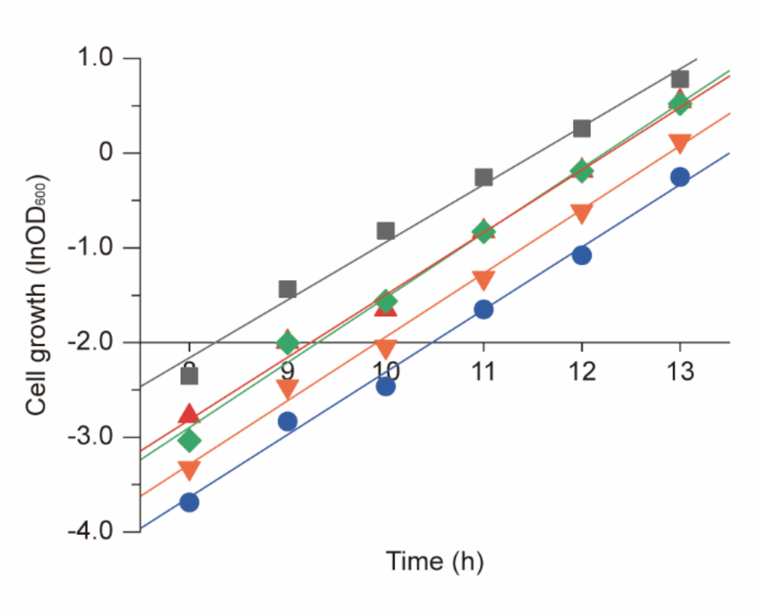

Figure S9. Linear-fitted plots from −4.0 to +1.0 of ln(OD_{600}) of (gray) native yeast, (blue) yeast[Fe^{3+}-BTC], (red) yeast[Fe^{3+}-P], (orange) yeast[Fe^{3+}-BTC]$_{anti\text{-}CD3/anti\text{-}CD28}$, and (green) yeast[$Fe^{3+}$-P]$_{anti\text{-}CD3/anti\text{-}CD28}$.
